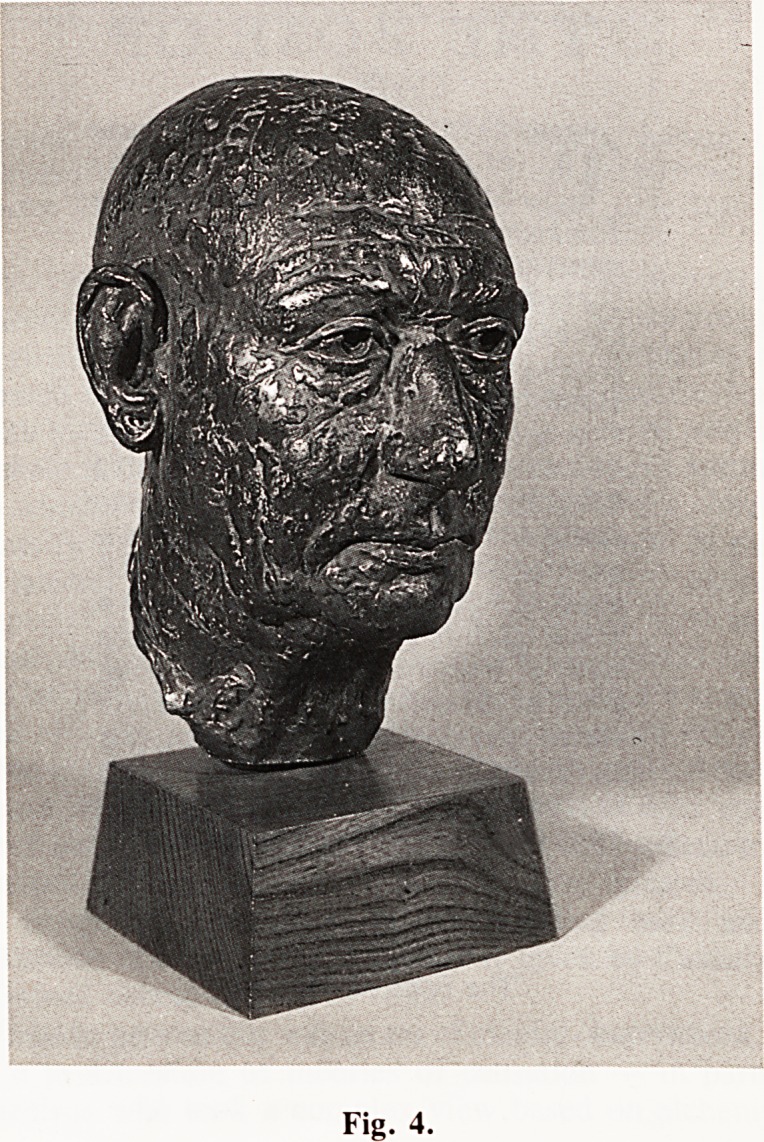# The Unwrapping of the Bristol Mummy

**Published:** 1991-12

**Authors:** J. Sluglett


### The Unwrapping of the Bristol Mummy, H7386

**J. Sluglett, OBE, MD**

This Egyption Mummy was given to the Bristol Museum by the Egypt Exploration fund in 1905. It came from Deir el Bahri, an ancient burial site on the left bank of the Nile near Luxor. During my survey of all the Bristol Mummies it was noticed that H7386 was deteriorating fast and might have to be destroyed. I had attended the Manchester symposium of Science in Egyptology in 1979 and this encouraged me to suggest that we should unwrap our mummy. Having obtained the consent of Bristol City Council, the owners of the mummy, it was left to me to organise a team of experts from professional colleagues known to me. This was duly done and the work was begun in the anatomy department of Bristol Medical school on April 1st 1981, and was completed on the 13th. Throughout the unwrapping I acted as commentator on a closed circuit television which was relayed to the main hall of the Bristol Museum where it was seen by an audience of over 25,000.

This was a carefully planned scientific project. One unique feature was a specially designed table divided by slats so that the dissectors could work from below without disturbing the mummy. A camera was inserted into the ceiling above the table and throughout the work, still photographs were taken as the dissection progressed. If funds could be found to convert these pictures on to a continuous film we would have a most fascinating piece of cinema. The mummy was never moved at any stage and all the work carefully plotted on special sheets. A great discovery was of beetles within the folds of the cloth but, much more fascinating was a laundry mark as it were, and this hieroglyph was deciphered to reveal the name Horemkenesi, a priest of Amun and supervisor of the temple building. From X-ray and other evidence it was clear that he was male aged about 60, possibly mildly diabetic, because of the evidence of Forrestier's disease in the vertebral column. He also had several dental abscesses which must have caused him a good deal of pain. The various members of the team have each contributed papers on what was discovered and there are also numerous pictures and a video of the whole project. Some of the latter has been on Points West and some of the papers in scientific journals but unfortunately, there is no complete book on the subject. There is also a special display of the project in the museum and a reconstruction of the head which has been made by Mr. E. Pascoe.

Scissors were used once only, to divide the large single covering which enclosed the mummy, all the wrappings were dissected slowly and carefully with scalpel and forceps. Each limb was wrapped separately, much of the linen was intact but some had been eaten away by insects which had entered before the elbalming process was completed. Some parts of the lower limbs had also perished in this way. The most fascinating moment for me came when the head and face was exposed and I found myself gazing at a dulled pair of grey eyes over a time span of three thousand years.

Someday, when funds permit or some benefactor is found, a book will be published, it ought to be as this is a very unusual piece of work and well deserving of being made into a permanent record. All the material is there.

**Fig. 1.**

Before the start of the project, April 1st 1981.

**Fig. 3.**

**Fig. 2.**

This picture I find fascinating. You are looking at a knot tied 3000 years ago. We have preserved this.

**Fig. 4.**

## Figures and Tables

**Fig. 1. f1:**
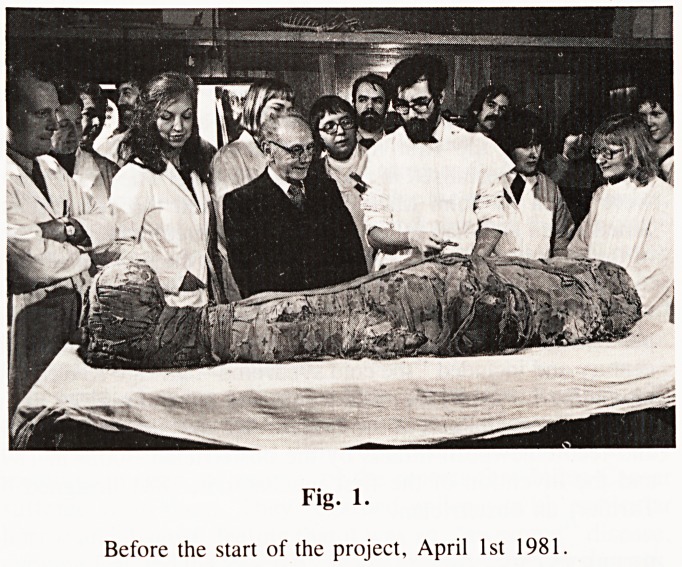


**Fig. 2. f2:**
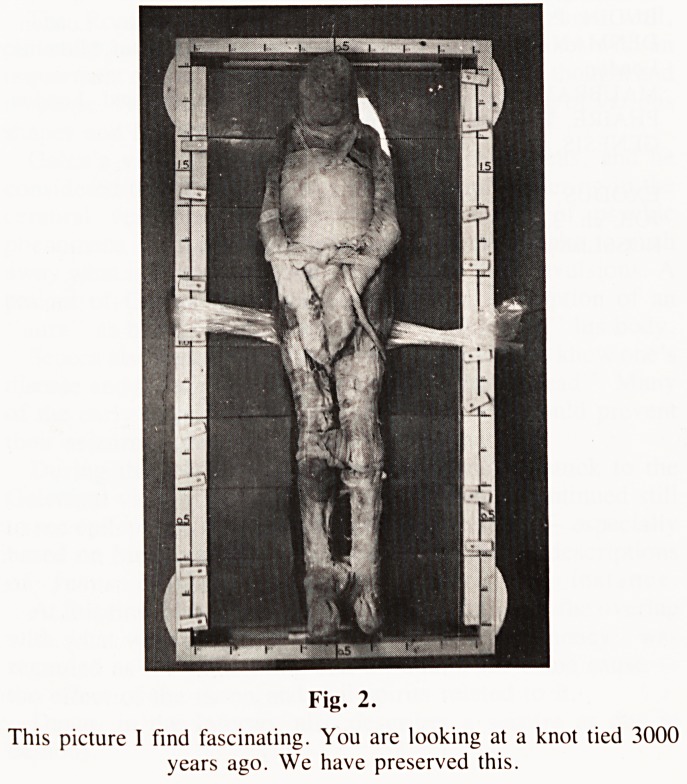


**Fig. 3. f3:**
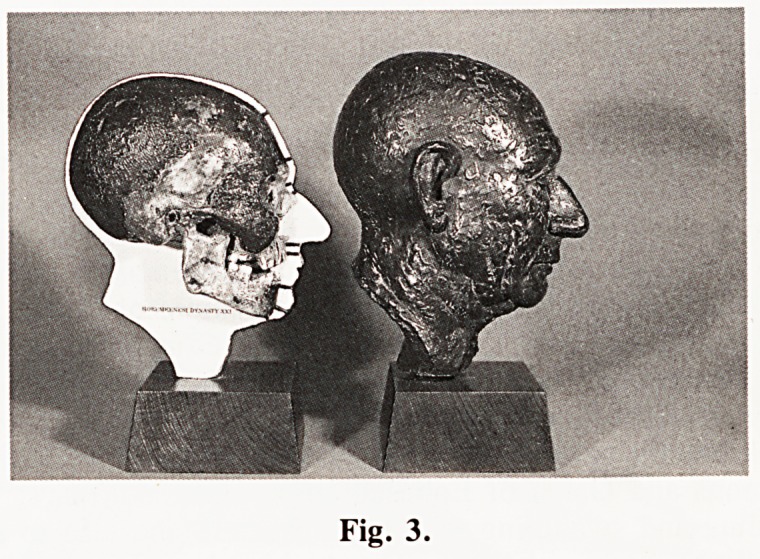


**Fig. 4. f4:**